# Bioresorbable Scaffold Use in Coronary Chronic Total Occlusions: A Long-Term, Single-Center Follow-Up Study

**DOI:** 10.3390/medicina60081233

**Published:** 2024-07-30

**Authors:** Dace Sondore, Ieva Briede, Matiss Linde, Karlis Trusinskis, Inga Narbute, Sanda Jegere, Aigars Lismanis, Indulis Kumsars, Karlis Grikis, Uldis Strazdins, Andrejs Erglis

**Affiliations:** 1 Pauls Stradins Clinical University Hospital, LV-1002 Riga, Latvia; dace.sondore@stradini.lv (D.S.); uldis.strazdins@stradini.lv (U.S.); 2 Faculty of Medicine and Life Sciences, University of Latvia, LV-1004 Riga, Latvia

**Keywords:** bioresorbable scaffolds, chronic total occlusion, percutaneous coronary intervention, coronary artery disease

## Abstract

*Background and Objectives:* Percutaneous coronary intervention (PCI) of chronic total occlusion (CTO) is often associated with longer total stent length. Our aim was to evaluate the long-term safety and effectiveness of bioresorbable scaffold (BRS) implantation in CTO to avoid using a full metal jacket. *Materials and Methods:* We conducted a single-center prospective longitudinal case study including 34 patients who underwent PCI of CTO with at least one BRS and drug-eluting stent (DES) implantation (*n* = 27) or BRS-only at the Latvian Centre of Cardiology between 2016 and 2018. Quantitative coronary angiography (QCA) and intravascular ultrasound were performed during the index procedure and long-term follow-up. *Results:* Of 34 patients with a mean age of 60.6 ± 9.5 years, 76.5% were male. The most common CTO artery was the right coronary artery (73.5%, *n* = 25). The median length of occlusion was 23.0 mm (interquartile range (IQR) = 13.9–32.7), with a total mean BRS/DES length of 49.6 ± 20.4 mm. During the median follow-up of 5.6 years (IQR = 5.0–5.9), the primary endpoint of target vessel re-occlusion occurred in 5.9% (*n* = 2) of patients. Target lesion revascularization (TLR) was performed in 35.3% (*n* = 12) of patients, with a mean time to TLR of 62.5 (95% confidence interval (CI), 53.9–71.2) months. Through QCA, there was a statistically significant increase in median residual diameter stenosis (20.1–31.4%, *p* < 0.01) and residual length of stenosis (5.2–7.1%, *p* = 0.04) compared with the index procedure. *Conclusions:* Our study demonstrates that BRS is a safe and feasible option for PCI of CTO, allowing for the avoidance of long segment stenting and ensuring long-term patency of the coronary artery.

## 1. Introduction

Chronic total occlusion (CTO) represents a challenging and complex subtype of coronary artery disease, characterized by Thrombolysis in Myocardial Infarction (TIMI) grade 0 flow and estimated occlusion duration of greater than or equal to 3 months [1]. The treatment of CTOs is a critical aspect of interventional cardiology, aimed at restoring coronary blood flow, relieving symptoms of ischemia, improving patient prognosis, and enhancing quality of life [2]. CTO PCI is proven to reduce anginal symptoms compared to optimal medical therapy, but the reduction in major adverse cardiac events remains uncertain, according to data from large randomized studies [3,4].

CTOs have been extensively studied using percutaneous coronary intervention (PCI) with drug-coated balloons or drug-eluting stents (DESs) [5,6,7,8,9]. The advancements in techniques and tools for CTO PCI, such as non-invasive and invasive imaging, have led to success rates of up to 80–90% [10]. The introduction of bioresorbable scaffolds (BRSs) has brought a new dimension to the percutaneous treatment paradigm [11]. BRSs offer several potential advantages over their metal and balloon counterparts, including the temporary provision of mechanical support to the vessel with the ability to dissolve and be absorbed after a certain period of time, thereby restoring the natural vasomotion and physiological responsiveness of the artery [12].

BRSs are particularly appealing in the context of CTOs because they can reduce the burden of permanent material in the coronary arteries, which is often considerable given the length and complexity of these occlusions. Many studies have assessed the safety and outcomes of BRSs in CTOs, but the main limitation has been the relatively short follow-up time [13,14,15]. Therefore, our study fills this gap by evaluating the long-term outcomes and safety of BRSs in CTO lesions. 

## 2. Materials and Methods

### 2.1. Study Design

This was a single-center prospective cohort study performed at the Latvian Centre of Cardiology, Pauls Stradins Clinical University Hospital. Patients were enrolled in the study between March 2016 and November 2018. We enrolled patients at least 18 years of age with stable angina or silent ischemia and single chronic CTO with TIMI 0 flow of at least 3 months duration. Major exclusion criteria were acute coronary syndrome within 1 month, life expectancy of less than 2 years, vessels with a reference diameter less than 2.5 mm or equal and higher than 4.0 mm, the presence of a contraindication to DESs and BRSs, and dual anti-platelet therapy. The evaluation employed quantitative coronary analysis (QCA) and intravascular ultrasound (IVUS) as invasive imaging tools. According to the protocol, clinical and angiographic follow-up was conducted at 12 months and 5 years post implantation to ensure a comprehensive understanding of the long-term safety and feasibility of bioresorbable scaffold implantation for CTO. Due to the COVID-19 pandemic, the follow-up visits scheduled for 2021 were postponed to 2022. The protocol was approved by the Ethics Committee of the Research Institute of Cardiology and Regenerative Medicine, University of Latvia for Clinical and Physiological Research, Drug and Pharmaceutical Product Clinical Investigation (Approval Nr. 01-290114). Informed written consent was obtained from all patients. 

### 2.2. Bioresorbable Scaffolds and Revascularization

In this study, we employed Magmaris (Biotronik AG, Bülach, Switzerland) and Absorb (Abbott Vascular, Santa Clara, CA, USA) scaffolds. Magmaris is primarily composed of a refined, slow-degrading proprietary magnesium alloy coated with a polylactic acid (PLLA) polymer that also includes sirolimus. The magnesium alloy allows for faster degradation and absorption within 9 to 12 months, which is significantly quicker compared to other BRSs [16]. Absorb is made from a poly-L-lactic acid (PLLA) polymer and features a poly-D, L-lactide (PDLLA) coating, which contains the antiproliferative drug everolimus. The complete absorption of the Absorb scaffold occurs over approximately 36 to 42 months, during which the scaffold supports the vessel before gradually being resorbed [16].

**Figure 1:**
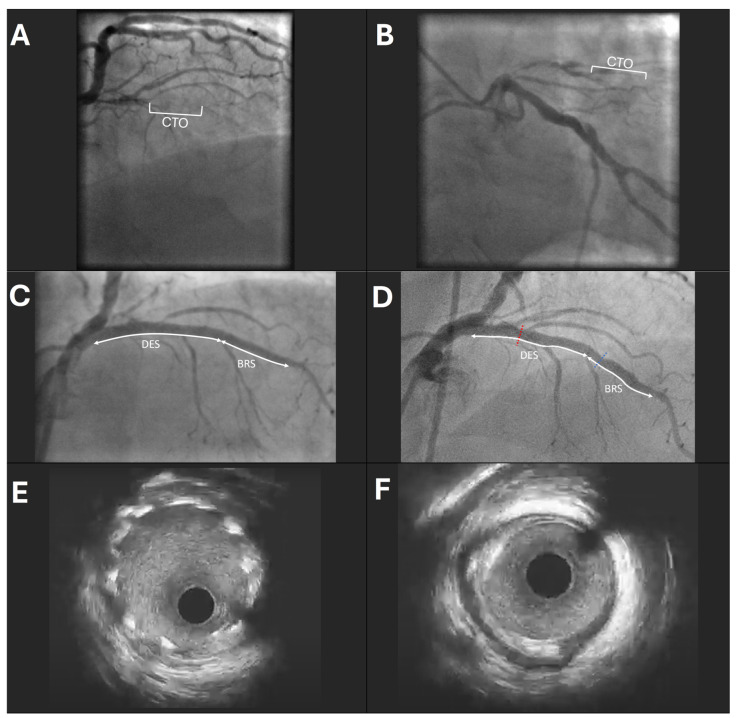
Representative case example of a patient with chronic total occlusion (CTO) in the left anterior descending artery (LAD) (**A**,**B**); (**C**) result of index percutaneous coronary intervention after implantation of 2 drug-eluting stents (DESs) and 1 bioresorbable scaffold (BRS) in a total length of 55 mm; (**D**) follow–up after six years; the patient is free from any major adverse cardiac events; the red dotted line (in **D**) represents the intravascular ultrasound (IVUS) cross-section of the DES without significant neointimal hyperplasia (**E**); the blue dotted line (in **D**) represents the IVUS cross-section of the BRS where the scaffold is fully dissolved (**F**).

The most common revascularization approach was the implantation of a DES in the proximal part of the lesion with the following 5 mm overlap with the BRS (Figure 1). In fewer cases, only a BRS was implanted. The treated segment with bioresorbable scaffolds and drug-eluting stents, including 5 mm proximal and 5 mm distal to the implanted device, was referred to as the target lesion. 

### 2.3. Study Endpoints

The primary endpoint was target vessel re-occlusion, defined as TIMI (Thrombolysis in Myocardial Infarction) grade 0 flow of a previously revascularized coronary artery, including thrombosis. The secondary endpoint was set as target lesion failure (TLF), defined as cardiac death, myocardial infarction, and target lesion revascularization (TLR). TLR was defined as a repeated PCI of the target lesion due to restenosis or other complications of the target lesion. Target vessel revascularization (TVR) was defined as a reintervention of the target vessel. The definitions used in the study are according to the Academic Research Consortium [17]. The patent artery was defined as persistent patency with TIMI 3 flow in the target vessel during the angiographic long-term follow-up.

### 2.4. Statistical Analysis

Continuous variables are presented as the mean ± standard deviation (SD). The quantitative variables were described by the median (Me) and interquartile range (IQR) when the data did not follow a normal distribution. Normality was assessed using both visual inspection of a normal probability plot and the Shapiro–Wilk test. Subsequently, a non-parametric Wilcoxon signed-rank test for non-normally distributed data and the parametric paired sample *t*-test for normally distributed data were performed. The Kaplan–Meier analysis was used to analyze the time-to-event data. All statistical analyses were conducted using IBM SPSS Statistics 22.0, and a significance level of *p* < 0.05 was chosen to indicate statistical significance.

## 3. Results

### 3.1. Patient Characteristics

Out of 34 patients with a mean age of 60.6 ± 9.5 years, the study predominantly included male patients (76.5%). Most patients had arterial hypertension (73.5%), and many had a history of myocardial infarction (47.1%) and prior PCI (73.5%). The median number of significant coronary lesions was 2.0 (IQR = 1.0–3.0). The median follow-up time was 5.6 years (IQR = 5.0–5.9). The characteristics of the population are represented in Table 1.

### 3.2. Procedural Characteristics

Most CTOs were in the right coronary artery (73.5%); the median Japanese-CTO score was 2.0 (IQR = 2.0–3.0). CTO complexity was classified as easy (J-CTO of 0) in 1 patient (2.9%), intermediate (J-CTO of 1) in 6 patients (17.6%), difficult (J-CTO of 2) in 13 patients (38.2%), and very difficult (J-CTO of ≥3) in 14 patients (41.1%). The antegrade approach was the most common method of PCI (67.6%). Various stent combinations were used, with the most common being one DES and one BRS implantation; for 20.6% (*n* = 7) of patients, only one BRS was implanted. The median length of occlusion was 23.0 mm (IQR = 13.9–32.7), and the total stent length was 49.6 ± 20.4 mm (seeTable 2).

### 3.3. Quantitative Coronary Angiography

QCA was performed for 34 patients at the index procedure and 31 patients at follow-up. There was a statistically significant increase in the residual diameter stenosis (20.1 (IQR = 14.9–26.7) % vs. 52.6 (42.7–65.5) %, *p* < 0.01) and area stenosis (36.1 (IQR = 27.5–46.2) % vs. 52.6 (IQR = 42.7–65.5] %, *p* < 0.01) at follow-up, indicating progression in lesion stenosis, but the average increase in stenosis was around 10%, indicating a persistent coronary artery lumen (see Table 3).

### 3.4. Intravascular Ultrasound

During the angiographic follow-up, IVUS was performed in 19 patients due to the operator’s decision. Table 4 represents detailed measurements of lumen and vessel areas and diameters. 

### 3.5. Follow-Up and Clinical Outcomes

The median length of follow-up was 5.6 [(QR = 5.0–5.9) years. Angiographical follow-up was performed in 91.2% of patients (*n* = 31). Three patients did not undergo follow-up angiography. One patient had terminal heart failure with a left ventricular assist device implanted, the second patient died due to gastric cancer, and the third patient was lost to follow-up. The primary endpoint of target vessel re-occlusion was observed in 5.9% (*n* = 2) of patients, and in both cases, occlusion occurred within the DES. One patient who underwent DES and Absorb implantation of the occluded right coronary artery experienced ST-elevation myocardial infarction two years post index procedure caused by DES thrombosis. He underwent successful revascularization; the target vessel was patent during long-term follow-up at six years. Another patient experienced asymptomatic re-occlusion of the DES without myocardial infarction, and optimal medical treatment was recommended without revascularization. During the long-term angiographic follow-up, 96.8% of CTO arteries were patent (30 out of 31). 

As a secondary endpoint, TLF was observed in 35.3% (*n* = 12) of patients during the follow-up due to one case of DES thrombosis, six cases of scaffold restenosis (three for Magmaris; three for Absorb), and three cases of DES restenosis. One patient had restenosis within the DES/BRS segment, and one patient had restenosis in the distal edge of the lesion where dissection was left after the index PCI. Another patient experienced progression of coronary artery disease in the proximal part of the target vessel. Therefore, TVR was performed in 38.2% (*n* = 13) of patients (see Table 5).

The Kaplan–Meier survival analysis showed a significant duration of efficacy for bioresorbable scaffolds in treating chronic total occlusions with a mean time to TLF and TLR of over 62.5 (95% confidence interval (CI), 53.9–71.2) months. As for TVR, the Kaplan–Meier survival analysis showed a decline over time, indicating cumulative occurrences of TVR at a mean of 60.8 (95% CI, 51.8–69.7) months. 

### 3.6. Laboratory Findings

Blood analysis was available for all 34 patients and during follow-up for the 31 patients who underwent angiography. There was a significant reduction in total cholesterol level (3.8 (IQR = 3.3–4.5) mmol/L vs. 3.3 (IQR = 3.0–3.5) mmol/L, *p* = 0.005), indicating effective lipid management, possibly due to medication adjustments, as lipid-lowering therapy was reassessed after the procedure. Changes in lower creatinine and glomerular filtration rate were also noted (See Table 6).

## 4. Discussion

Our study shows that BRSs are a feasible and safe option for the treatment of CTOs. Most commonly, one DES and one BRS (Absorb or Magmaris) were implanted in the CTO artery, avoiding a full metal jacket. The primary endpoint of target vessel re-occlusion occurred in only 5.9% (*n* = 2) of patients due to one DES restenosis and one DES thrombosis, indicating effective long-term follow-up results. Of the 31 patients with angiographic follow-up, the target vessel was patent during follow-up in 96.8% (*n* = 30) of patients. The secondary endpoint of TLF was observed in 35.3% (*n* = 12) of patients, the same as TLR. TLF and TLR had a mean survival time to an event of 62.5 (95% CI, 53.9–71.2) months. A higher rate of TVR of 38.2% (*n* = 13) suggests that the disease may progress in other parts of the CTO artery that require revascularization. This could be due to the natural progression of coronary artery disease, and the survival time free from TVR was at a mean of 60.8 (95% CI, 51.8–69.7) months. 

BRSs provide several advantages over traditional metallic stents, including the ability to offer temporary vascular support and then dissolve, allowing the artery to regain its natural structure and functionality [16]. In recent years, the use of BRSs in practice has been questioned regarding their safety and efficacy. For example, the ABSORB III trial [18] emphasized these concerns, as target vessel myocardial infarction was more frequent with BRSs compared with everolimus-eluting stents (EESs) (8.6% vs. 5.9%; *p* = 0.03) and scaffold thrombosis (ScT) was significantly higher in BRS patients (2.3% vs. 0.7%; *p* = 0.01). The Food and Drug Administration raised this safety concern and sales of Absorb BRS were discontinued [19]. The pathophysiology of these adverse effects could be related to incomplete endothelialization or early dismantling of the scaffold which can promote thrombosis, and the structural integrity of the BRS can also be compromised over time as the material degrades, leading to potential scaffold disruption and late adverse events such as vessel restenosis or late thrombosis [16].

Interestingly, procedural aspects were analyzed in detail in the ABSORB trials [20], which showed that implantation in properly sized vessels (reference vessel diameter between 2.25 mm and 3.75 mm) independently predicted freedom from TLF through the first year (hazard ratio (HR): 0.67; *p* = 0.01) and over three years (HR: 0.72; *p* = 0.01). Also, aggressive pre-dilation was an independent predictor of reduced ScT rate between the first and third year (HR: 0.44; *p* = 0.03). Optimal post-dilation was independently associated with a reduced rate of TLF between the first and third year (HR: 0.55; *p* = 0.05) as well. These technical BRS implantation factors were strictly fulfilled in our study.

Later in the ABSORB IV trial, despite improved implantation technique, a slightly higher five-year TLF rate of 17.5% compared to 14.5% for cobalt chromium EESs was demonstrated, with a statistically significant difference (*p* = 0.03). This increased risk was primarily observed during the first three years post implantation, coinciding with the scaffold’s bioresorption period. The study also noted a non-significant difference in device thrombosis rates within five years, occurring in 1.7% of BVS patients versus 1.1% of CoCr-EES patients (*p* = 0.15) [21].

Regarding the bioresorbable magnesium scaffold (Magmaris), the BIOSOLVE-IV study demonstrated BRSs to be both safe and effective in treating coronary artery disease, with a 12-month TLF rate of 4.3%, a device success rate of 97.3%, and a procedure success rate of 98.9%. The study found a low scaffold thrombosis rate of 0.5%, with most events linked to early cessation of antiplatelet therapy [22]. The results align well with the BIOSOLVE-II and BIOSOLVE-III trials, which also showed favorable safety and performance outcomes with no definite or probable scaffold thrombosis [23]. 

So far, the choice of scaffold, whether polymer based like Absorb or metal based like Magmaris, impacts clinical outcomes and procedural success. However, CTOs are more complicated coronary artery disease lesions that have not been studied as extensively as simple lesions. In our study, we found TLF to be related to an equal count of Magmaris and Absorb restenosis.

In studies assessing the efficacy and safety of DESs in de novo CTO lesions during 5-year follow-up, 6.3% TLR and 7.1% TVR were recorded [24] with similar results in another study with a 4-year follow-up [4]. Comparably, in the ABSORB-CTO study [25] of 35 lesions, a rate of only 3% for TLF and TLR was recorded in the 3-year follow-up, but a rate of 11.4% was recorded for target vessel re-occlusion. Another study demonstrated that the Absorb BRS is non-inferior to EESs after 12 months of follow-up [15]. We assessed TLF and TLR in 35.3% of cases, while only two re-occlusions were noted in our study. The higher TLF and TLR rates in our study may be due to more complex lesions and procedures compared to those in the ABSORB-CTO study. According to the J-CTO score in our study, most of the lesions were difficult (38.2%) or very difficult (41.1%) to examine, while in the ABSORB study, the CTOs were mostly intermediate (48.6%) or easy (25.6%) to examine. As a result, the retrograde approach was more often used in our study (32.4% vs. 14.3%) [26]. Scheduled angiographic follow-up may increase the rate of revascularization due to oculostenotic reflex more than ischemia-driven indication for PCI [27]. Another important factor is the lack of intravascular imaging in PCI guidance. Our earlier study involving the use of Absorb in treating left main distal bifurcation lesions demonstrated that the use of intravascular ultrasound for optimization was a protective factor against major adverse coronary events [28]. Intravascular imaging also plays a crucial role in CTO treatment. IVUS offers deeper tissue penetration compared to optical coherence tomography (OCT). It also has the ability to operate without contrast and is effective in real-time imaging and stent optimization in the CTO treatment [29]. In contrast, OCT is less suited for the broad requirements of CTO PCI, where flexibility and a comprehensive view of the vessel are paramount [29]. CTO-IVUS and AIR-CTO studies showed that IVUS-guided CTO revascularization notably decreased major cardiovascular events during follow-up [30,31]. In our study, IVUS was employed during follow-up, showing a minimal lumen area of 5.35 ± 1.2 mm^2^ and a mean lumen area of 8.83 ± 1.9 mm^2^. QCA demonstrated that the residual diameter increase was around 10%. This showcases the durability of the BRS CTO approach.

### 4.1. Limitations

While we have described the long-term efficacy and safety of BRSs in CTO lesions, our study has some limitations. The study was conducted at a single center, which may limit the generalizability of the findings to other populations or settings. With only 34 patients, the sample size is relatively small, which could affect the statistical power and the ability to detect more minor effects or differences. It is important to note that enrollment occurred at a time when concerns about BRS safety had arisen, which led to the withdrawal of Absorb. We did not have a comparative group using drug-eluting stents only, which limits the ability to directly compare the effectiveness and safety of BRS to other established treatment options. IVUS was performed only during follow-up, which limits the assessment of differences from the index procedure. The different absorption rates of the scaffolds (Magmaris vs. Absorb) and their impact over time were not extensively analyzed, which could influence long-term outcomes like scaffold integrity and vessel reactivity.

### 4.2. Suggestions for Future Research

As one of the bioresorbable scaffolds used in this study has been taken off the market (Absorb; Abbott Vascular) and the other (Magmaris, Biotronik) has the third generation available, future studies are needed to explore the potential advantages of recently developed bioresorbable vascular scaffolds in the treatment of CTO. Larger randomized trials with DESs as a control group should be undertaken to evaluate the superiority of new BRSs over currently available DESs.

## 5. Conclusions

This study demonstrated that BRSs are a safe and feasible option for PCI of CTOs, allowing for the avoidance of long segment stenting and ensuring long-term patency of the coronary artery. Despite some limitations, the primary endpoint of target vessel re-occlusion was 5.9% with a high target vessel patency rate of 96.8% during the angiographic follow-up, indicating that BRSs are effective in keeping CTO arteries open over the long term. More than one-third of patients had TLF and TLR. This suggests that while BRSs are generally successful, there are cases where the lesion may fail over time. The slightly higher rate of TVR suggests that the disease may progress in other parts of the CTO artery, necessitating further interventions. Overall, while the study demonstrates promising results for the use of BRSs in treating CTOs, the noted limitations highlight the need for further research with larger, multicenter trials to confirm these findings and better understand the long-term benefits and risks associated with BRSs in CTO PCI.

## Figures and Tables

**Table 1:** Characteristics of the study population.

Parameter	N = 34
Age, years	60.6 ± 9.5
Gender:	
Male	26 (76.5)
Female	8 (23.5)
Arterial hypertension	25 (73.5)
Atrial fibrillation	3 (8.8)
Chronic heart failure, NYHA class:	
NYHA I	4 (11.8)
NYHA II	11 (32.4)
NYHA III	1 (2.9)
Chronic kidney disease	5 (14.7)
Diabetes mellitus	4 (11.8)
Hypercholesterolemia	14 (41.2)
History of myocardial infarction	16 (47.1)
Prior PCI	25 (73.5)

The results are the mean ± standard deviation or number (%). N: number; NYHA: The New York Heart Association Classification; PCI: percutaneous coronary intervention.

**Table 2:** Index procedural characteristics.

Parameter	N = 34
CTO vessel:	
Right coronary artery	25 (73.5)
Left descending artery	8 (23.5)
Circumflex artery	1 (2.9)
J-CTO score	2.0 [2.0–3.0]
7 French sheath size	27 (79.4)
Number of predilatation balloons used per lesion	2.0 [2.0–3.0]
Cutting balloon predilatation	34 (100.0)
Post-dilatation	34 (100.0)
Approach to CTO:	
Antegrade	23 (67.6)
Retrograde	11 (32.4)
Length of occlusion, mm	23.0 [13.9–32.7]
Type of stents implanted:	
1 DES and 1 BRS	18 (52.9)
2 DES and 1 BRS	7 (20.6)
1 BRS only	7 (20.6)
2 BRS and 1 DES	2 (5.9)
Number of DESs implanted	1.0 [1.0–1.0]
Number of BRSs implanted	1.0 [1.0–1.0]
1 Absorb	21 (61.8)
2 Absorb	2 (5.9)
1 Magmaris	11 (32.4)
Total length of stents and/or scaffold, mm	49.6 ± 20.4

The results are the mean ± standard deviation, median (IQR: interquartile range), or number (%). CTO: chronic total occlusion; BRS: bioresorbable scaffold; DES: drug-eluting stent; J-CTO score: the Japanese chronic total occlusion score; N: number.

**Table 3:** Quantitative coronary angiography analysis of the target lesion.

Parameter	Index (N = 34)	Follow-Up (N = 31)	*p*-Value
Residual diameter stenosis, %	20.1 [14.9–26.7]	31.4 [24.4–42.0]	<0.01
Residual area stenosis, %	36.1 [27.5–46.2]	52.6 [42.7–65.5]	<0.01
Length of residual stenosis, mm	4.5 [2.8–6.8]	5.9 [3.9–9.4]	0.04

The results are the median (IQR: interquartile range). N: number.

**Table 4:** IVUS characteristics of the target lesion during follow-up.

Parameter	N = 19
Target segment length, mm	43.4 ± 19.1
Maximal lumen area, mm^2^	13.3 ± 4.2
Minimal lumen area, mm^2^	5.35 ± 1.2
Mean lumen area, mm^2^	8.83 ± 1.9
Maximal vessel area, mm^2^	22.2 ± 6.5
Minimal vessel area, mm^2^	10.3 ± 3.6
Mean vessel area, mm^2^	15.7 ± 4.4
Maximal lumen diameter, mm	4.1 ± 0.6
Minimal lumen diameter, mm	2.6 ± 0.3
Mean lumen diameter, mm	3.3 ± 0.36
Maximal vessel diameter, mm	5.3 ± 0.8
Minimal vessel diameter, mm	3.54 ± 0.6
Distal lumen area, mm^2^	7.79 ± 1.7
Distal lumen diameter, mm	3.1 ± 0.3
Distal vessel area, mm^2^	13.7 ± 4.4
Distal vessel diameter, mm	4.1 ± 0.35
Proximal lumen area, mm^2^	9.6 ± 2.9
Proximal lumen diameter, mm	3.5 ± 0.5
Proximal vessel area, mm^2^	18 ± 5.7
Proximal vessel diameter, mm	4.7 ± 0.8
Neointimal hyperplasia, %	21.3 [17.7–22.4]

The results are the mean ± SD (standard deviation) of the median (IQR: interquartile range). N: number.

**Table 5:** Clinical outcomes at long-term follow-up.

Parameter	N = 34
Target vessel re-occlusion	2 (5.9)
Cardiac death	0 (0)
Myocardial infarction	1 (2.9)
BRS thrombosis	0 (0)
DES thrombosis	1 (2.9)
Target lesion failure	12 (35.3)
Target vessel failure	13 (38.2)
Target lesion revascularization	12 (35.3)
Target vessel revascularization	13 (38.2)

The results are the number (%). BRS: bioresorbable scaffold; DES: drug-eluting stent. N: number.

**Table 6:** Measurement of blood biochemistry values.

Parameter	Index (N = 34)	Follow-Up (N = 31)	*p*-Value
Total cholesterol, mmol/L	3.8 [3.3–4.5]	3.3 [3.0–3.5]	0.005
LDL cholesterol, mmol/L	1.9 [1.6–2.3]	1.8 [1.3–1.9]	0.39
HDL cholesterol, mmol/L	1.1 [0.9–1.2]	1.0 [0.8–1.3]	0.11
Triglycerides, mmol/L	1.4 [0.9–2.4]	1.5 [1.2–2.0]	0.62
Creatinine, µmol/L	77.0 [71.0–91.0]	83.5 [76.5–91.5]	0.01
GFR, mL/min/1.73 m^2^	97.0 [76.0–102.0]	88.5 [69.5–93.0]	0.01
Glucose, mmol/L	5.8 [5.1–6.9]	5.8 [5.2–6.7]	0.56

The results are the median (IQR: interquartile range). GFR: glomerular filtration rate; N: number; LDL: low-density lipoprotein; HDL: high-density lipoprotein.

## Data Availability

Data are contained within the article.
